# A New Approach for Treatment of Woman With Absolute Uterine Factor Infertility: A Traditional Review of Safety and Efficacy Outcomes in the First 65 Recipients of Uterus Transplantation

**DOI:** 10.7759/cureus.12772

**Published:** 2021-01-18

**Authors:** Iana Malasevskaia, Ahmed A Al-Awadhi

**Affiliations:** 1 Obstetrics and Gynecology, Private Clinic, Sana'a, YEM; 2 Research, California Institute of Behavioral Neurosciences & Psychology, Fairfield, USA; 3 Anaesthesiology, German-Yemeni Hospital, Sana'a, YEM

**Keywords:** uterus transplantation, post-operative complications, infertility, in vitro fertilization ivf, mayer-rokitansky-küster-hauser (mrkh) syndrome, transplantation, absolute uterine factor infertility (aufi), hysterectomy

## Abstract

Uterine transplantation restores fertility in women with absolute uterine factor infertility and allows the opportunity to conceive, experience gestation, and acquire motherhood. The number of cases being performed is increasing dramatically, with detailed outcomes from 65 cases now available. Pregnancies achieved through uterus transplantation and following in vitro fertilization (IVF) are associated with an increased risk for further mother and newborn babies. This traditional review is focused on the safety and efficacy features of the treatment. However, it is associated with significant risk, with approximately one-quarter of grafts are removed because of complications. Uterine transplantation is realizable in women with uterine factor infertility but is associated with a significant complication risk. The risk of the procedure and gestational and delivery complications deserve important consideration before receiving such treatments. Nevertheless, these observations are preliminary and should be revised after a larger series of data are published.

## Introduction and background

Historically, society believes that for girls to become "real mothers," they must carry a pregnancy themselves. The desire for pregnancy is so strong that some women are willing to risk their health to have a baby [1]. 

Absolute uterine factor infertility (AUFI), which refers to infertility that is completely attributable to the uterine absence (congenital or surgical) or an abnormality (anatomic or functional), affects approximately one in 500 women of childbearing age, or 1.5 million women worldwide [2]. Uterine factor infertility affects thousands of women worldwide, caused by congenital Müllerian malformations, such as in the Mayer-Rokitansky-Küster-Hauser (MRKH) syndrome, or acquired as in the cases of women suffering from Asherman's syndrome, pregnancy-interfering myomas, or hysterectomies [2,3]. 

Motherhood choices traditionally offered to women with AUFI spin around two options: adoption or surrogacy. Adoption provides legal motherhood, while a surrogacy agreement confers genetic motherhood and, after adoption, legal motherhood to infertile mothers. During this process, multiple medical and legal issues may arise. Maternal surrogacy is forbidden in many countries, including France, Germany, Bulgaria, Croatia, Estonia, Finland, Hungary, Italy, Lithuania, Portugal, Slovakia, Australia, Holland, Spain, Sweden, and Norway. However, there is no legislation or regulation in other countries, leading to conflict between the involved parties [2]. 

The evolution of uterine transplantation (UTx) was primarily motivated by the potential to help women suffering from the discrepancy between procreative ability and reproductive aspirations because of AUFI. Although this procedure is associated with greater risk from multiple major surgeries and exposure to immunosuppressant drugs, UTx is the only option that allows women with AUFI the opportunity to become pregnant and give birth to genetically correlated offspring themselves [4]. 

Women who are inspired to pursue uterus transplantation take risks from immunosuppression, a high‐risk pregnancy, and at least three surgical procedures. The first procedure includes allotransplantation, then the caesarian section to deliver the child (and a second section if a second child is desired), and a graft hysterectomy after the delivery [5]. 

Steps required pre/post the uterus transplantation procedure

The transplant process to successful birth fluctuates from person to person but can take approximately two to five years for many participants [5-7]. It includes: 

1*. *Medical evaluation of donor and recipient [5-7]. 

2.Embryo generation: Before the surgery, a woman generates embryos through in vitro fertilization (IVF). During this process, she should take fertility drugs to produce eggs removed from her ovaries and fertilized outside of her body and frozen for later use [5-7]. 

3.Transplantation: The uterus is removed from a donor during a three-hour operation and surgically placed into the recipient in a six-hour operation. The recipient starts taking immunosuppressive drugs to prevent rejection of the transplant. These medications should be taken while the transplant is in place and during pregnancy [5-7]. 

4.Pregnancy: Several months or a year after the transplant surgery, one of the recipient's embryos will be placed into the uterus. If implantation of the embryo is successful, the recipient will become pregnant. Both baby and mother's health are monitored closely at frequent prenatal care visits with a high-risk obstetrician, known as a maternal-fetal medicine specialist [5-7]. 

5. Delivery: The baby is born as close to term as is possible via a planned cesarean section. If the pregnancy has gone without any complications, and the patient wants one more child, the uterus is left, and immunosuppression medications are continued. Another embryo transfer can be attempted after approximately six months after delivery [5-7]. 

6.Uterus removal: When childbearing is complete, the transplanted uterus must be removed, and immunosuppressive medications should be stopped [5-7]. 

**Figure 1:**
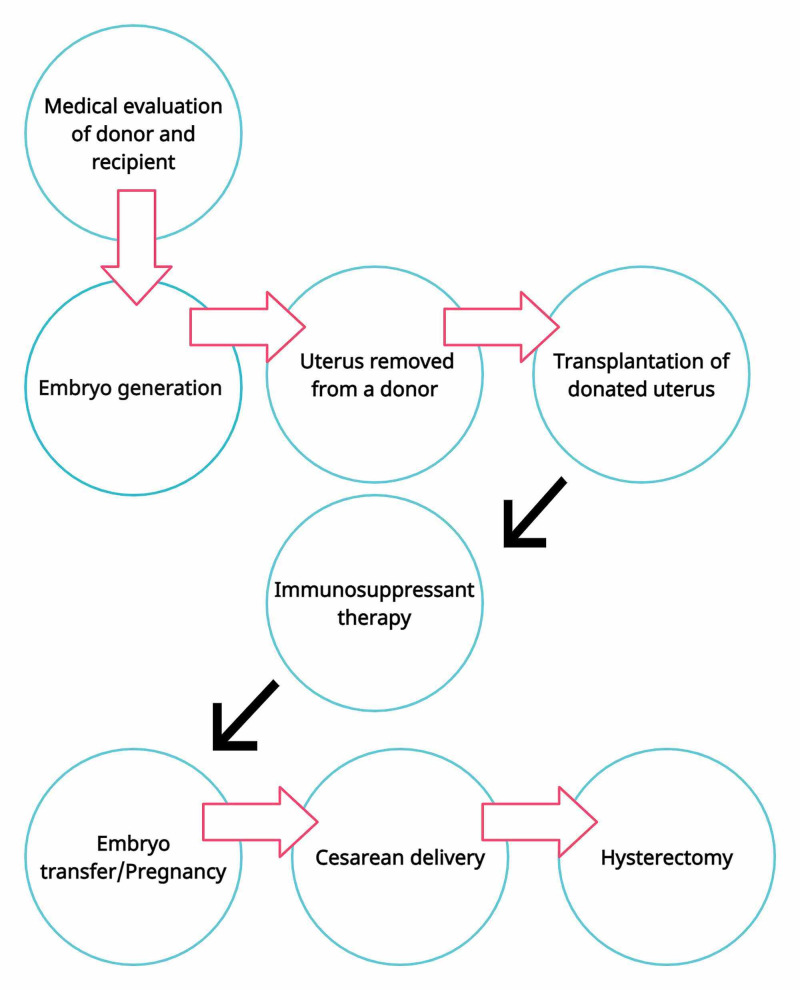
How uterus transplantation works. The process from the first medical evaluation, transplant to a successful birth. (Figure created by Malasevskaia I.)

 Figure 1 describes in short the steps followed during uterus transplantation procedures. 

Possible complications following uterus transplantation 

All surgeries have risks and benefits; therefore, it's important to fully consider them before deciding whether the procedure is appropriate for the patient or not. As with any operation, uterus transplantation also puts the donor and the recipient at risk. 

The Czech Republic team reported vaginal stenosis, vesicovaginal fistula, cytomegalovirus, and herpes infection in recipients [8]. The donors undergo a surgical procedure similar to radical hysterectomy; therefore, the risk of urinary tract complications is always a concern. In a recipient with MRKH syndrome, there is always a risk for fistula formation because the newly formed vagina (neovagina) has to be detached from the bladder, which leads to the possibility of a fistula between these organs. Additionally, there is a higher risk of thrombosis in anastomotic vessels in UTx because narrower vessels are used compared with those in other organ transplantation. Measures for preventing immunosuppressant-induced infection and pancytopenia are also required, and caution is needed regarding rebleeding from un-ligated capillaries and the vaginal cuff after reperfusion. Insufficient hemostasis may cause postoperative intraabdominal complications or retroperitoneal hematoma [8]. 

**Figure 2:**
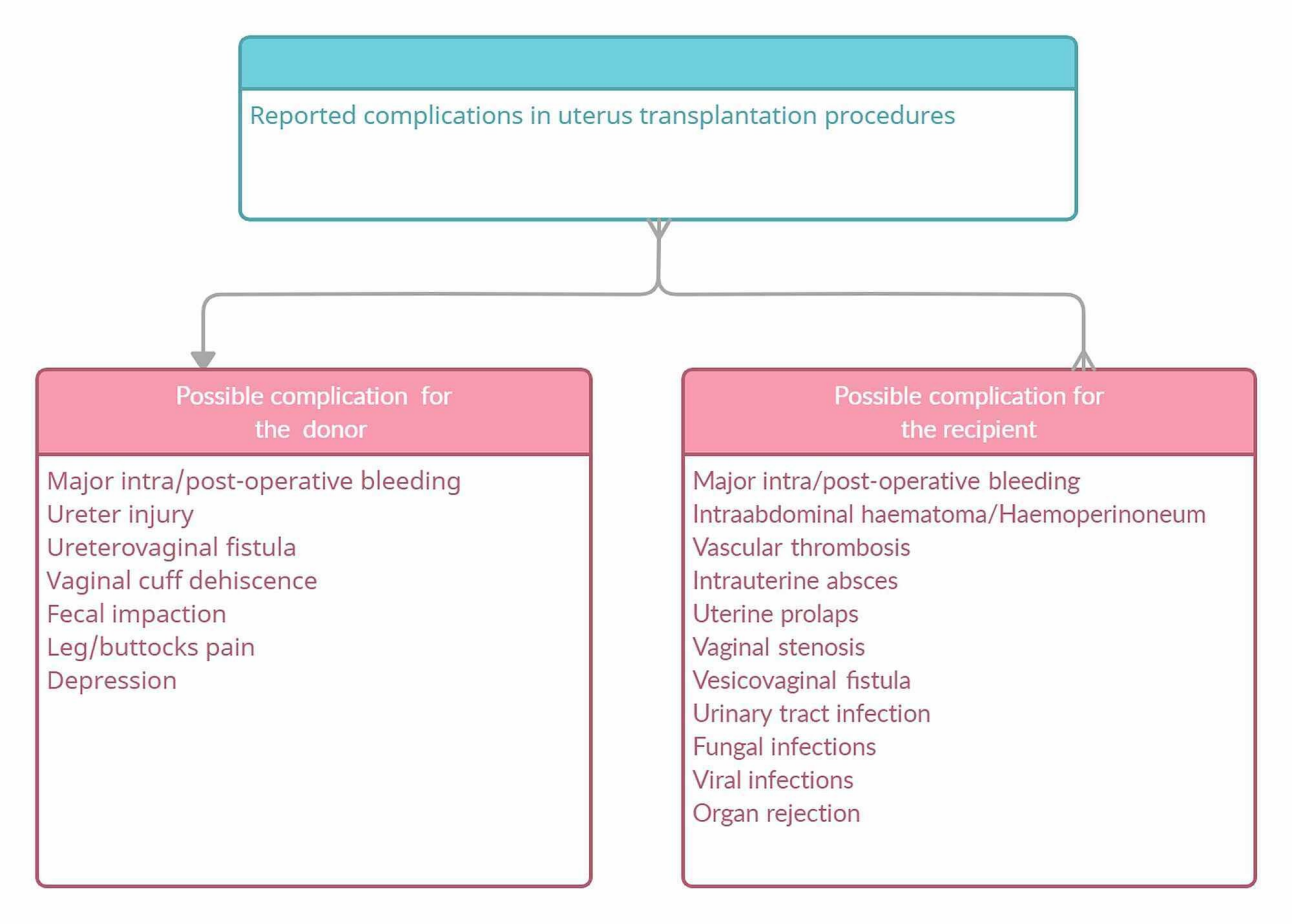
Reported complications faced by donors and recipients during and after uterus transplantation procedures. (Image created by Malasevskaia I.)

The likelihood of rejection for a transplanted uterus is still unclear; however, rejection has been detected in cervical biopsy for many transplanted uteruses after UTx. Some patients develop rejection before and during pregnancy, which is managed with immunosuppressant medication [8]. Figure 2 summarizes reported complications faced by donors and recipients during and after the procedures needed for uterus transplantation. 

This review article aimed to analyze the first uterus transplantations done in the past 20 years. Additionally, we tried to investigate the complications faced by specialists following this sophisticated procedure. 

## Review

Methods 

An extensive literature search on Google, Google Scholar, PubMed, and non-PubMed indexed journals was performed. Literature databases were searched to identify the information available about uterus transplantation, using the following terms: "uterus transplantation," "uterus transplantation and complications," "first uterus transplantation," "babies born after uterus transplantation." The full text of the articles eligible for our study was reviewed and included in the review. We only included articles written in the English language and were done on human models, while studies on animals were excluded. Only the most relevant research articles are included. 

The first uterus transplantation, Saudi Arabia 

The first human uterine transplantation was performed on April 6, 2000. The recipient was a 26-year-old female who lost her uterus six years earlier due to delivery complicated with post-partum hemorrhage; the donor was a 46-year-old patient with multiloculated ovarian cysts who underwent a hysterectomy to preserve tissue and vascular integrity. The donor uterus was connected to the recipient's vaginal vault with additional fixation using a shortened uterosacral ligament. The uterine arteries and veins were connected to the external iliac arteries and veins using segments of the great saphenous vein [9]. 

Immunosuppression was maintained by oral cyclosporine A, azathioprine, and prednisolone. An acute rejection episode was treated and controlled on the ninth day with anti-thymocyte globulin (ATG). The transplanted uterus responded well to combined estrogen-progesterone therapy, with endometrial proliferation up to 18 mm, which resulted in withdrawal bleeding upon cessation of the hormonal therapy. Unfortunately, 99 days after transplantation, vascular thrombosis developed, and hysterectomy was necessary. Macro- and microscopic histopathological examination showed acute thrombosis in the uterine body vessels, which resulted in infarction. Both fallopian tubes were unchanged, with no evidence of rejection. Inadequate uterine support caused acute vascular occlusion, which led to probable tension, torsion, or kinking of the connected vascular uterine grafts [9]. 

This first human case, although a failure, inspired researchers around the world to initiate new studies on uterus transplantation, immunosuppression, rejection, and pregnancy outcome. 

Uterus transplantation, Turkey 

The second uterus transplantation, and the first from a deceased donor, was performed in Turkey in 2011. The recipient was a 22-year-old woman diagnosed with MRKH syndrome, and the donor was a 22-year-old nulliparous woman with brain death after a car accident. From past medical history, she also underwent vaginal reconstruction surgery with jejunum segment two years before transplantation [10,11]. 

The retrieval procedure resembled radical hysterectomy and dissection of vascular pedicles, including hypogastric, uterine artery, and ovarian vessels, from surrounding tissues. Additionally, the bladder peritoneum's anterior reflection was dissected and included within the retrieved uterus. The anastomosis was performed between the recipient's external iliac vessels and the hypogastric artery pedicle of the donor's uterus. By the 20th month of the postoperative period, the recipient did well with no signs of rejection, continuing to menstruate. Six IVF cycles were attempted, with one chemical and three missed abortion pregnancies (two of which had fetal cardiac activity) [11]. 

They elected to use a deceased donor as its main advantage is the possibility of achieving longer and enhanced vascular grafts within a significantly short time [11]. 

This was the first heart beating pregnancy following deceased donor transplantation, an important step in uterus trial attempts. Although these pregnancies ended with missed abortion, they were still direct evidence of the viability of a uterus retrieved from a deceased donor. On the other hand, the donor's nulliparous state with unproven fertility may have adversely affected pregnancy chances. Moreover, previous vaginal reconstruction with an intestinal flap might have impeded the implantation process, similar to the adverse effect of hydrosalpinx on in vitro fertilization (IVF) cycles. Although the intestinal neovagina no longer performed a fecal storage function, normal bacterial flora may have diminished endometrial receptivity and abnormal expression of factors related to implantation [11]. 

Based on Daily Sabah News, in her 28th week of pregnancy, Derya Sert gave birth on June 4, 2020, to a healthy baby boy, with the help of plastic surgeon Dr. Ömer Özkan of Akdeniz University in Antalya province. After successful birth, the uterus was removed [12]. 

Uterus transplantation, Sweden 

The first clinical trial of uterus transplantation was started in Sweden in 2013 and involved nine live donor procedures. This trial included nine recipients (27-38 years of age), with eight having MRKH syndrome. In contrast, the ninth patient had a radical hysterectomy because of cervical cancer seven years before UTx. The donors were in seven cases genetically related, and two were non-genetically related. The donors' age varied from 37 to 62 years, with five being postmenopausal. Surgical success was established in eight of nine, but one graft was removed three days postoperatively due to bilateral arterial and venous thrombosis. Another recipient developed an intrauterine infection two months after UTx. Despite treatments with antibiotics and surgical drainage, a hysterectomy had to be performed three and a half months later when septicemia developed. Seven of nine patients had regular menstruations during the initial year and were scheduled for embryo transfer 12 months post-transplantation [13]. 

The first uterus transplantation was done for a 35-year-old woman with a congenital absence of the uterus (Rokitansky-Mayer syndrome). The uterus donor was a living, 61-year-old, two-parous woman. The first menstruation occurred 43 days after uterus transplantation, and she continued to menstruate at regular intervals afterward at the median of 32 days. One year after transplantation, the patient underwent her first single embryo transfer, which was successful and resulted in a viable pregnancy. Triple immunosuppression (tacrolimus, azathioprine, and corticosteroids) was given and continued during pregnancy. She had three mild rejection incidents, one of which occurred during pregnancy which was managed by corticosteroid treatment. At 31 weeks of pregnancy and five days, the patient was admitted with preeclampsia, and a cesarean section was done because of abnormal cardiotocography. A healthy baby boy with normal birth weight for gestational age (1,775 g) and with appearance, pulse, grimace, activity, respiration (APGAR) scores of 9, 9, 10 was born [14]. Additionally, two more successful births followed within that trial in November 2014 [15]. 

The first uterus transplant involving surgeon-operated robots was performed in Sweden in October 2017 without any complications. The robot-assisted procedure involved making a one-centimeter incision in the abdomen of the donor, who was the recipient's mother. Two robot arms performed the procedure, managed by a human surgeon. The uterus was removed through that incision and directly transferred to the recipient via open surgery carried out by humans. Ten months after transplantation, the recipient became pregnant via IVF. The pregnancy went without any complications, and a baby boy was delivered by cesarean section [16]. 

Additionally, another uterus transplant was done in December 2019 in Sweden, for the first time from a deceased donor. The recipient was a 30-year-old woman, and the donor was a woman who had previously given birth and was of fertile age at the time of her sudden death. Ten months after surgery, attempts will be started to make the woman pregnant [17]. 

Since 2014, when the first baby was born, another eight babies, including three sibling pairs, were born in Sweden; the latest birth took place in January 2020 [17]. However, Dr. Mats Brännström reported to *Transplantation* in June 2020 that >10 babies were born in Sweden from uterus transplant recipients [18]. 

Uterus transplantation, China

China's first human uterus transplant was done in 2015; the recipient was a 22-year-old woman, and the donor was her mother of 43 years [19]. The procedure was done by robotic-assisted laparoscopic uterus retrieval from the mother [20]. The frozen embryo was successfully implanted in the patient's womb on June 13, 2018, after the fifth attempt. During early pregnancy, there were three episodes of vaginal bleedings, which were treated with corticosteroids and tacrolimus [19]. A cesarean section was conducted at gestational week 33 + six days due to uterine contraction suggesting imminent labor. A healthy baby boy (2,000 g) with APGAR scores of 10, 10, 10 was delivered. The uterus was kept for a possible second pregnancy [20]. Additionally, two transplantations were done in China using a living donor, but full details are unknown [21]. 

Uterus transplantation, USA 

The first uterus transplantation in the USA was performed in February 2016 in Cleveland Clinic. The recipient was a 29-year-old mother of five adopted children, and the donor was a deceased woman in her 30s. Unfortunately, on March 7, the patient began bleeding, and doctors discovered that an artery connected to supply the uterus was damaged and had no choice but to remove the organ. The cause was revealed to be *Candida* infection from the transplanted uterus [22]. 

Doctors at Baylor University Medical Center, USA, performed four uterus transplantations in September 2016, but only one has proven successful. This was the first living-donor uterine transplant performed in the USA [23]. In November 2017, a 29‐year‐old woman who had a successful procedure at Baylor University Medical Center in September 2016, born without a uterus because of MRKH syndrome (Type 1), gave birth to a healthy baby boy [24]. 

Two important modifications from the previously reported live births characterized how this uterus transplant was done. First, the transplanted uterus had only the utero-ovarian vein as venous outflow; second, the time from transplantation to embryo transfer was shortened from prior protocols, allowing for shorter exposure to immunosuppressant drugs by the patient, which minimized the risk for potential adverse effects from these medications [23]. 

From 2016 to 2020, Baylor University Medical center reports that 20 uterus transplants were performed. Twenty women were enrolled in the clinical trial, with a median age of 29.7 years, with 10 in the first and second phases. All but two recipients had a congenital absence of the uterus. Eighteen recipients received a uterus from living donors and two from deceased donors. In phase one, 50% of recipients had a technically successful uterus transplant, compared to 90% in phase two [25]. The institution performed 18 living donor hysterectomies for uterus transplantation, five cases using a robot-assisted technique and 13 cases by an open laparotomy technique [26]. Uterus removal was required due to vascular thrombosis in four recipients, graft ischemia in two recipients, and postoperative hemorrhage in one patient [27]. 

One of the uterus transplantation recipients from the Baylor University Medical Center clinical trial gave birth to the second baby girl in December 2020. She was the fifth woman to undergo uterus transplantation from a living donor in the clinical trial. The procedure went without any complication, and the first baby girl was born in 2018 [28]. 

To date, 20 women have undergone uterus transplantation at Baylor University, and 13 babies were born as part of Baylor's uterine transplant program [28]. 

In October 2015, Cleveland Clinic began an interventional clinical trial to perform 10 uterine transplantations from October 2015 to October 2023. This study has seven phases: primary and secondary screening, medical evaluation, IVF, transplantation, embryo transfer, pregnancy/delivery, and follow-up after delivery. Women will undergo deceased donor uterine transplantation after IVF [29]. Although a failure followed the first one, this did not stop the team from performing following transplantations [22]. 

Since Cleveland Clinic started the clinical trial, the team has completed eight uterus transplants; six were successful, and two resulted in hysterectomies soon after transplantation [30]. The first baby girl was born in 2019 at Cleveland Clinic; she was the first baby in North America delivered by a mother who received a deceased donor's uterus transplant. In her mid-30s, the mother was one of the women participating in the groundbreaking research trial involving 10 women with uterine factor infertility [31]. In March 2020, the second time, Cleveland Clinic has delivered a baby boy from a uterus that was transplanted from a deceased donor. The uterus was transplanted in early 2019, and in late 2019, the recipient, who was 31, became pregnant through in vitro fertilization [30]. 

**Figure 3:**
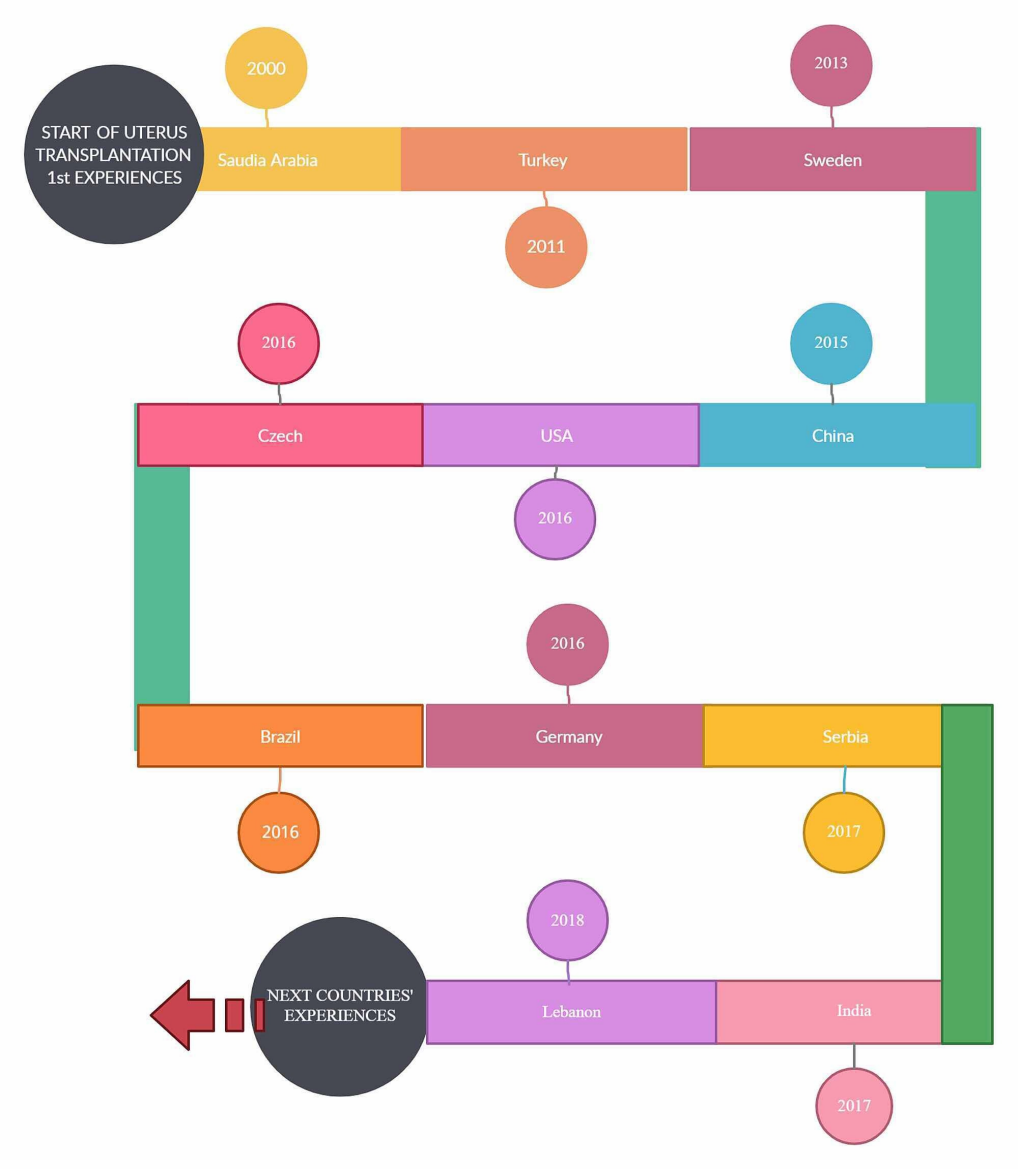
Countries that first started to apply uterus transplantation. (Image created by Malasevskaia I. )

Figure 3 schematically shows countries that started performing uterus transplantation from 2000 to 2020. 

Uterus transplantation, Czech Republic 

The Czech UTx trial was established in July 2015 after approval by the Ministry of Health, Czech Republic. The first UTx of that trial was performed in April 2016 with a living donation from mother to daughter, and the first deceased donor (DD) UTx was performed in June 2016. As of November 2017, another seven uterine transplantations had been performed, in total, nine transplantations, four from deceased and five from living donors (LDs) [32]. 

All the recipients were healthy, and all of them had MRKH syndrome. Donors aged 18 to 60 years old were used, LDs underwent a thorough examination, having a normal uterus, without a history of cancer, diabetes, hypertension, or any other serious disease, including infectious diseases. DDs had a normal uterus and no history of cancer, diabetes, or other chronic or infectious diseases. A maximum of four deliveries, including one cesarean section, were accepted for uterine donation. Both LDs and DDs had transvaginal ultrasound examination, hysteroscopy, and Pap smear before UTx. In two of the recipients, the uterus was transplanted from nulliparous DDs. Mild rejection episodes occurred seven times in three recipients, while moderate rejection occurred only once. Two uterine grafts were removed on day seven (DD) and day 15 (LD) after the UTx because of vascular thrombosis discovered by regular clinical examination and transabdominal Doppler ultrasound [32]. 

One graft from a surgically successful DD UTx was removed on post-transplant month seven due to Herpes simplex virus‐2 (HSV‐2), which destroyed the uterine cavity and cervical canal. Histopathologic examination of the graft showed fibrotic obliteration of the cervical canal with signs of rejection in the myometrium [32]. 

Partial postoperative stenosis of the uterine‐vaginal (or vaginal‐vaginal) anastomosis developed in three recipients, which were found between the first and second months after UTx. The stenosis was most likely caused by the discrepancy between the large uterine cervix and the narrow and tough vaginal vault. Spontaneous menstruation started within five to 14 weeks post-transplant in all six recipients with a functional uterus [32]. 

In August 2016, a 26‐year‐old woman with MRKH syndrome was the fourth person worldwide who received a uterine transplant from a deceased donor. The donor was a 24‐year‐old brain‐dead nulliparous woman. The fifth frozen embryo transfer was successful, which resulted in a clinical pregnancy, and three weeks later, an intrauterine gestational sac containing an embryo with a heartbeat was detected. However, the pregnancy resulted in missed abortion [33]. 

Uterus transplantation, Brazil 

The first worldwide case of a live birth following uterine transplantation from a deceased donor happened in Brazil. A 32-year-old woman with MRKH syndrome underwent uterine transplantation in São Paulo, Brazil, in September 2016, from a 45-year-old woman who died of subarachnoid hemorrhage. The donor had three previous vaginal deliveries [34]. 

The recipient had satisfactory postoperative recovery and was discharged after eight days of observation in the hospital. Immunosuppressive drugs were begun with prednisolone, thymoglobulin, and continued via tacrolimus and mycophenolate mofetil (MMF). Five months post-transplantation, azathioprine replaced MMF [34].

First menstruation started 37 days post-transplantation, which was regular (every 26-32 days). Pregnancy occurred after the first embryo transfer seven months post-transplantation. No abnormalities on blood flow velocity waveform were detected by Doppler ultrasound of uterine arteries, fetal umbilical, middle cerebral arteries, or any fetal growth impairments during pregnancy. No rejection episodes were noted after transplantation or during gestation. Cesarean delivery occurred on December 15, 2017, near week 36 of pregnancy. The baby girl weighed 2,550 g at birth, with APGAR scores of 9, 10, 10 and developing normally seven months postpartum. The uterus was removed, and immunosuppressive therapy was suspended [34]. 

Uterus transplantation, Germany 

In October 2016, the first UTx was performed in Germany. In total, four pairs of uterus recipients and living donors underwent complete UTx procedures between October 2016 and January 2019. Recipients were from 23 to 35 years, and all had type 1 MRKHS. Donor ages were between 32 to 56 years, with only one postmenopausal donor. All donors gave birth to at least two children, to a maximum of four deliveries [35]. 

The donor surgery involved harvesting the uterus with its blood vessels without the ovaries; the Fallopian tubes were removed to avoid extrauterine pregnancies. The arterial vasculature involved the deep uterine artery bilaterally with a segment of the internal iliac arteries, and the venous vasculature connected to the uterine graft to be harvested one or two deep uterine veins on both sides, connected to a segment of the internal iliac vein, and/or the proximal parts of the utero-ovarian branch, divided proximally to the inlet of the ovarian veins, to preserve the ovaries in situ [35]. 

Recipient surgery comprised preparation by dissection of the vaginal vault and the external iliac vessels. Anastomoses were performed end to the side from the segments of the graft’s deep uterine arteries to the recipient’s external iliac arteries and the segments of the graft’s internal iliac veins to the recipient’s external iliac veins. In recipients with a thin deep uterine vein on one side, the proximal part of the utero-ovarian vein was used for an additional venous outflow, either by anastomosis onto the graft segment’s internal iliac veins or directly onto the external iliac veins. After proper reperfusion, the recipient’s vagina was opened, and vaginal-vaginal anastomosis was performed. The uterus was affixed to the sacro-uterine and round ligaments, while the bladder's peritoneum of the graft was sutured with the recipient’s bladder [35]. 

There were no complications encountered intra- or postoperatively, except in one recipient, who showed impaired arterial flow on the right side intraoperatively, necessitating re-anastomosis. Suturing the right uterine artery end to side onto the external iliac artery in addition to a segment of the internal iliac artery with adjacent proximal vesical artery were performed. Immunosuppressive (IS) regimens for UTx were adopted from kidney transplantation [35]. 

Induction therapy was begun with anti-thymocyte globulin (ATG) for three days, and the parallel start of a triple-drug IS regimen with tacrolimus, MMF, and prednisolone. MMF was replaced after approximately six months with azathioprine (AZA) for at least three to six months given a planned pregnancy. To improve individual tolerability, tacrolimus was replaced by cyclosporin. According to the donor’s and recipient’s cytomegalovirus (CMV) status, prophylaxis with valganciclovir for three to six months was done. Additionally, cotrimoxazole for six months for *Pneumocystis jirovecii *prophylaxis was prescribed. Two mild rejection episodes were observed in two recipients, and one recipient developed a CMV infection seven months post-surgery, which was successfully treated with valganciclovir [36]. 

After the third single-embryo transfer, one recipient got pregnant, which was attempted at month 17 but ended in an early miscarriage at eight weeks and four days of pregnancy. However, following the next ovarian restimulation and oocyte fertilization procedures, she and another recipient underwent a fresh single-blastocyst transfer, resulting in successful intrauterine pregnancies in both [35].

As a result, the two mothers (25 and 26 years old) gave birth to the 15th and 17th children globally, the first baby in March and a second in May 2019 [36]. The first baby's delivery occurred at week 35 + one day after preterm rupture of membranes in the absence of any signs of infection or labor. At week 36, the second delivery took place as scheduled via cesarean section [35]. 

Uterus transplantation, Serbia 

In March 2017, in Serbia at University Children's Hospital in Belgrade, a 38-year-old woman received a uterus transplant from her twin sister. The transplant was conducted in Belgrade by the Swedish medical team of Dr. M. Brännström, the pioneer of the uterus transplant technique, and assisted doctors from the Serbian hospital and physicians from Brigham Women's Hospital and Harvard Medical School, both in Boston, Massachusetts [37]. 

According to Bologna Today, the uterus extraction lasted 10 hours, and the transplant lasted five hours without any complications. The sister who donated her uterus already has three children. After the transplant, the woman traveled to Stockholm to perform an IVF procedure (the post-transplantation management is not mentioned). Following an IVF procedure, she gave birth to a healthy baby boy at Sant' Orsola Hospital in Bologna, Italy [37]. 

Uterus transplantation, India 

On May 18, 2017, a 21-year-old woman born with a congenital absence of the uterus became the first person to undergo a womb transplant in Pune, Inda. A team of doctors transferred her mother's uterus to her using a minimally invasive procedure. It took four-and-a-half hours to retrieve the uterus from the 41-year-old woman through minimally invasive keyhole surgery, medically known as laparoscopic surgery. In contrast, womb transplant surgery took nine hours [38]. 

On May 19, 2017, a second uterine transplant was performed by the same team. The second recipient of a uterus was a 24-year-old woman, and the donor was her 45-year-old mother. The same procedure was successfully followed [38]. 

The third successful uterine transplant was conducted in January 2018 by the same Galaxy Care Hospital team in Pune. While the first two womb transplants were completed in approximately nine hours, the third was completed in six hours. All three womb transplants at Galaxy Care Hospital were performed through laparoscopic surgery [38]. 

In April 2018, another two successful uterus transplantation procedures were conducted in India at an urban, private, tertiary care hospital. Two patients, ages 30 and 24 years, diagnosed with MRKH syndrome, underwent LD uterine transplants; donors were their mothers of 48 and 47 years old, respectively. The IVF protocols and the results are not mentioned in the paper [39].

Retrieval of organs was done through mini-laparotomy and laparoscopic intervention. The ovarian veins were used as outflow channels, which allowed to avoid the challenges involved in dissection along the internal iliac vein while harvesting the donor internal iliac artery reduced the tension on the vascular anastomosis [39]. 

Oral immunosuppression with tacrolimus and mycophenolate mofetil was started 24 hours before surgery and continued postoperatively. Everolimus was commenced a month later to prevent rejection and allow for the decrease of the tacrolimus dose. Intravenous dexamethasone was administered postoperatively and gradually tapered to be replaced by oral prednisolone. Oral valganciclovir and cotrimoxazole were added on postoperative day one to prevent opportunistic infections. Subcutaneous heparin for three weeks, replaced by aspirin, was used for antithrombotic prophylaxis. Both recipients started menstruating within two months of surgery [39]. 

In October 2018, the first baby girl was born via cesarean section at Galaxy Care Hospital in Pune, India. The baby was born 17 months after the uterine transplant, at 34 weeks of gestation. The uterus transplant was done on May 19, 2017. The baby was delivered prematurely as the mother's blood pressure began to rise suddenly, and the fluid around the baby had started to reduce [40]. 

Uterus transplantation, Lebanon 

The first uterus transplantation in Lebanon was conducted by a team of doctors from Lebanon and Sweden on June 11, 2018 [41]. The recipient was a 24-year-old woman with MRKH syndrome, and the donor her 50-year-old multiparous mother. Both uterus retrieval and transplantation were performed by laparotomy. Donor's uterus was isolated with uterine arteries and veins, round ligaments, sacro-uterine ligaments, and bladder peritoneum [42]. 

The immunosuppression protocol consisted of induction by tacrolimus, azathioprine, thyroglobulin, and methylprednisolone. Thyroglobulin was given daily for five days, methylprednisolone was given perioperatively, and the prednisolone (per os) p.o. was given for six days. Maintenance immunosuppression was by daily administration of tacrolimus and azathioprine to maintain adequate immunosuppression. Mycamine was given as antifungal prophylaxis, daily valganciclovir for six months was used as CMV prophylaxis, and trimethoprim/sulfamethoxazole for prevention of *Pneumocystis jirovecci* pneumonia was given for three months [42]. 

The first menstruation occurred three weeks after UTx, which was regular afterward, from 28 to 30 days. The patient developed vaginal stenosis, which required manual dilations three times. Due to persistent anemia, the patient received erythropoietin two times a week and oral ferrous sulfate with vitamin B12 three months before embryo transfer due to low hemoglobin levels [42]. 

Embryo transfer was performed 10 months after uterus transplantation, which was successful and resulted in a positive pregnancy test after 14 days. Cesarean section was performed at 35 weeks gestation due to short uterus cervix and premature contractions registered on cardiotocography (CTG). A healthy female fetus was delivered 15 min after the skin incision [42]. 

Table 1 summarizes the outcomes and complications resulting from uterus transplantation in countries that have done the procedure. Based on published data, 65 UTx procedures were done from 2000 to 2020 in 11 countries; 16 (24.6%) were unsuccessful. 

**Table 1:** Outcomes of uterus transplantation based on published data. LD- living donor, DD- deceased donor, N/K- not known, UTx- uterus transplantation.

Country	Year of UTx		LD/DD	Reported complications of the recipients
Saudia Arabia	2000		LD	Inadequate uterine support caused acute vascular occlusion, thrombosis- hysterectomy was necessary.
Turkey	2011		DD	No complications were reported.
Sweden	Clinical trial started in 2013		9 LD	One graft was removed three days postoperatively due to bilateral arterial and venous thrombosis. Another recipient developed an intrauterine infection and sepsis two months after UTx- both needed hysterectomy
	2017		LD	No complications were reported.
	2019		DD	No complications were reported.
China	2015		LD	No complications were reported.
	N/K		LD	N/K
	N/K		LD	N/K
USA	Clinical trial started in 2016 (Cleveland Clinic)		8 DD	First UTx failed due to Candida infection found in donor’s uterus, which caused erosion of uterine artery and intraabdominal bleeding-hysterectomy was necessary. Additionally, another hysterectomy was necessary; the full information is N/K.
	The first clinical trial started in 2016 to 2018, second trial from 2018 to 2020 (Baylor University Medical Center)		18 LD 2DD	Uterus removal was required due to vascular thrombosis in four recipients, graft ischemia in two recipients, and postoperative hemorrhage in one patient.
Czech Republic	Clinical trial started in 2016		5 LD / 4 DD	Two uterine grafts were removed on day seven (DD) and day 15 (LD) because of vascular thrombosis. Another graft (DD) was removed on post-transplant month seven due to Herpes simplex virus‐2 (HSV‐2), which destroyed the uterine cavity and cervical canal. Partial postoperative stenosis of the uterine‐vaginal anastomosis developed in three recipients, which were found between the first and second months of the postoperative period.
Brazil	2016		DD	No complications were reported.
Germany	Clinical trial started in 2016		4 LD	One recipient showed impaired arterial flow on the right uterine side intraoperatively, necessitating re-anastomosis.
Serbia	2017		LD	No complications were reported.
India	2017		2 LD	No complications were reported.
	2018		3LD	No complications were reported.
Lebanon	2018		LD	Vaginal stenosis, which required manual dilations three times. Anemia.
In total			65 UTx (47LD/ 18DD)	16 uterus grafts were removed post-UTx due to complications.

Limitations of this review 

This review article is a traditional review and, therefore, does not follow the standard Preferred Reporting Items for Systematic Reviews and Meta-Analyses (PRISMA) guidelines for systematic reviews. 

Further research

Uterus transplantation is rapidly spreading worldwide and is starting to be recognized as a new assisted reproductive technology and transplantation technique. However, it is still at the experimental stage and has many medical, technical, and ethical issues. The uterus transplantation procedure is a constant risk for further mothers, starting with transplantation, an immunosuppressive status, and ending with the cesarean section and hysterectomy.

As observed, the babies born as a result of uterus transplantation should be born prematurely at less than 37 weeks of gestation, as specialists are concerned that as the uterus grows, this can affect the uterine vascular supply. Premature birth puts newborn babies at risk for apnea of prematurity, respiratory distress syndrome, infections or neonatal sepsis, intraventricular hemorrhage, necrotizing enterocolitis, retinopathy of prematurity, jaundice, and anemia. Some premature babies have to spend more time in a hospital’s newborn intensive care unit to get special medical care. Premature birth can also lead to long-term challenges for some babies, including intellectual and developmental disabilities. Long-term observation and control of babies born as a result of uterus transplantation from a living donor and a deceased donor are preferable, and case-control studies between babies born naturally and babies born after uterus transplantation.

Further studies should concentrate on the safest operation method to minimize the complications on the donor and recipient. Additionally, further research is needed on the immunosuppressive protocol, which is used before and during the pregnancy period. Long-term observation of the patients (recipients) after hysterectomy is demanded. As they have to have a big intervention which changes the anatomy of pelvic floor muscles, the direction of ureters, and place of the ovaries, these may predispose to pelvic organ prolapse and urinary tract infections with further kidney damage. Additionally, these procedures can affect the vascular supply of pelvic organs and lower limbs.

Until now, there is no clear information regarding how many weeks gestation a transplanted uterus can tolerate, newly formed vaginal vault, and deranged pelvic floor; further research is still needed. And is there any chance for a twin pregnancy? 

## Conclusions

Uterus transplantation is a new treatment approach of absolute uterine factor infertility, which has proceeded rapidly from an experimental procedure in animals to a successful clinical application. However, at this time, uterus transplantation should be considered a clinical experimental procedure until sufficient experiences have been collected from clinical trials expected to enroll during the next years. There is a significant clinical demand for uterus transplantation; therefore, it is expected that the number of procedures will increase.
